# Low expression of pro-apoptotic proteins Bax, Bak and Smac indicates prolonged progression-free survival in chemotherapy-treated metastatic melanoma

**DOI:** 10.1038/s41419-020-2309-3

**Published:** 2020-02-13

**Authors:** Cristiano Guttà, Arman Rahman, Claudia Aura, Peter Dynoodt, Emilie M. Charles, Elodie Hirschenhahn, Jesuchristopher Joseph, Jasper Wouters, Ciaran de Chaumont, Mairin Rafferty, Madhuri Warren, Joost J. van den Oord, William M. Gallagher, Markus Rehm

**Affiliations:** 1 Institute of Cell Biology and Immunology, University of Stuttgart, Stuttgart, Germany; 2 Oncomark Ltd., Nova UCD, Dublin, 4 Ireland; 3 UCD School of Biomolecular and Biomedical Science, UCD Conway Institute, University College Dublin, Dublin, 4 Ireland; 4 Pathology Diagnostics Ltd., Stirling House Business Centre, Waterbeach, Cambridge, UK; 5 Translational Cell and Tissue Research, KU Leuven (University of Leuven), Leuven, Belgium; 6 Department of Physiology and Medical Physics, Royal College of Surgeons in Ireland, Dublin, 2 Ireland; 7 Centre for Systems Medicine, Royal College of Surgeons in Ireland, Dublin, 2 Ireland; 8 VIB-KU Leuven Center for Brain & Disease Research, KU Leuven, Leuven, Belgium; 9 Department of Human Genetics, KU Leuven (University of Leuven), Leuven, Belgium; 10 Stuttgart Research Center Systems Biology, University of Stuttgart, Stuttgart, Germany; 11 Stuttgart Center for Simulation Science (SC SimTech), University of Stuttgart, Stuttgart, Germany

**Keywords:** Melanoma, Prognostic markers

## Abstract

Despite the introduction of novel targeted therapies, chemotherapy still remains the primary treatment for metastatic melanoma in poorly funded healthcare environments or in case of disease relapse, with no reliable molecular markers for progression-free survival (PFS) available. As chemotherapy primarily eliminates cancer cells by apoptosis, we here evaluated if the expression of key apoptosis regulators (Bax, Bak, Bcl-2, Bcl-xL, Smac, Procaspase-9, Apaf-1, Procaspase-3 and XIAP) allows prognosticating PFS in stage III/IV melanoma patients. Following antibody validation, marker expression was determined by automated and manual scoring of immunohistochemically stained tissue microarrays (TMAs) constructed from treatment-naive metastatic melanoma biopsies. Interestingly and counter-intuitively, low expression of the pro-apoptotic proteins Bax, Bak and Smac indicated better prognosis (log-rank p < 0.0001, p = 0.0301 and p = 0.0227 for automated and p = 0.0422, p = 0.0410 and p = 0.0073 for manual scoring). These findings were independently validated in the cancer genome atlas (TCGA) metastatic melanoma cohort (TCGA-SKCM) at transcript level (log-rank p = 0.0004, p = 0.0104 and p = 0.0377). Taking expression heterogeneity between the markers in individual tumour samples into account allowed defining combinatorial Bax, Bak, Smac signatures that were associated with significantly increased PFS (p = 0.0002 and p = 0.0028 at protein and transcript level, respectively). Furthermore, combined low expression of Bax, Bak and Smac allowed predicting prolonged PFS (> 12 months) on a case-by-case basis (area under the receiver operating characteristic curve (ROC AUC) = 0.79). Taken together, our results therefore suggest that Bax, Bak and Smac jointly define a signature with potential clinical utility in chemotherapy-treated metastatic melanoma.

## Introduction

Melanoma, an aggressive neoplasm originating from the malignant transformation of melanocytes, rapidly metastasises if not surgically removed at an early stage. Although novel and costly targeted treatment options and immunotherapies have significantly improved the management of metastatic disease^1–3^, patients in poorly funded healthcare environments still rely on chemotherapy as the primary first-line treatment. Likewise, chemotherapy remains in frequent use as a second- or last-line treatment option in otherwise refractory or in recurrent disease. Even though treatments based on the DNA-alkylating agent dacarbazine have been the chemotherapeutic standard of care for metastatic melanoma for > 30 years, chemotherapy may benefit only few patients^4,5^. The median survival of patients treated with dacarbazine-based chemotherapy lies in the range of 6–9 months^6–8^, with no reliable molecular markers available that would allow to identify those patients in which disease progression is substantially delayed and which therefore might have benefited from this treatment.

Apoptosis is the main cell death mechanism by which the body tries to eliminate transformed and therefore potentially cancerous cells. Apoptosis likewise is the primary cell death modality induced by dacarbazine and other DNA-alkylating agents. DNA alkylation induces the intrinsic apoptosis pathway, as was shown experimentally in various melanoma model systems^9,10^. Pro- and antiapoptotic Bcl-2 family members, such as Bax, Bak and Bcl-2, Bcl-xL, respectively, regulate the mitochondrial apoptosis signalling hub^11^. Activated Bax and Bak form pores in the outer mitochondrial membrane, leading to the release of pro-apoptotic factors, such as Smac, into the cytosol^12^. Subsequently, the execution phase of apoptosis is initiated, during which proteases such as initiator caspase-9 and effector caspase-3 are activated in an Apaf-1-dependent manner. These proteases then rapidly execute apoptotic death, but can be inhibited by the antiapoptotic protein XIAP, which itself is targeted by Smac^13^. Impaired apoptosis signalling is a hallmark of cancer^14^, based on which it is reasonable to assume that melanoma cells are highly apoptosis resistant. Indeed, experimental studies suggest that melanoma cells either are highly chemoresistant or acquire resistance and thereby evade apoptotic cell death^15,16^. However, it is less clear if perturbed expression of apoptosis regulators is indeed associated with patient prognosis in the clinical scenario. Various studies immunohistochemically assessed individual apoptosis regulators as potential protein biomarkers for melanoma progression and patient survival^17,18^. Unfortunately though, the majority of studies lack controls and validation information that would support the specificity of the used reagents and staining protocols. Not surprisingly, results obtained so far remained largely inconclusive or even contradictory^17^. Additionally, apoptosis regulators at key signalling hubs frequently act cooperatively and redundantly, so that it can be speculated that single molecule makers might not be sufficiently robust for clinical use.

In this study, we therefore assessed the expression of nine apoptosis regulators (Bax, Bak, Bcl-2, Bcl-xL, Smac, Procaspase-9, Apaf-1, Procaspase-3 and XIAP) in metastatic melanoma tissues by immunohistochemistry (IHC), using antibodies that passed rigorous validation. Interestingly, low expression of Bax, Bak and Smac associated with prolonged progression-free survival (PFS), a finding confirmed at transcriptional level in an independent cohort. Combining Bax, Bak and Smac expression with a pattern recognition approach allowed predicting individual patient PFS with high accuracy. Taken together, our results identified a putative combinatorial prognostic signature with potential clinical utility for chemotherapy-treated metastatic melanoma.

## Materials and methods

### Ethics approval and consent to participate

The use of the patient cohort was approved by the Medical Ethical Committee and Institutional Review Board (OG032) of the University Hospitals of KU Leuven (reference number ML10659) and by the UZ Leuven Biobank (reference number S56609).

### Antibodies

The following antibodies were used for immunoblotting and immunohistochemistry. A rabbit polyclonal beta Actin antibody (Santa Cruz Biotechnology; sc-81178); Apaf-1 (Cell Signalling; D5C3), Bak (Abcam; ab32371), Bax (Millipore; ABC11), Bcl-2 (Dako; MO887), Bcl-xL (BD labs; 610212), Procaspase-3 (Cell Signalling; 9662), Procaspase-9 (Cell Signalling; 9502), Smac (Cell Signalling; 2954), XIAP (BD labs; 610762).

### Cell culturing

For antibody validation, the following human cancer cell lines were used: A375, HCT-116, HCT-116 (Bax/Bak)^−/−^, HCT-116 Smac^−/−^, HCT-116 XIAP^o/−^, HeLa, Jurkat Casp-9^−/−^, MCF-7, PM-WK, Preyer, SK-Mel-94. Cell lines were obtained from ATCC, DSMZ or provided by colleagues (Professor Martin Leverkus, University of Heidelberg; Professor Richard Youle, National Institutes of Health, USA; Professor Richard Vogelstein, The Johns Hopkins University School of Medicine, USA; Professor Ingo Schmitz, University of Braunschweig, Germany; Professor Sebastian Wesselborg, University of Düsseldorf, Germany; Professor Maria Soengas, National Cancer Research Centre, Spain) and described before^19–24^. Cell lines were cultured in RPMI-1640 medium (Sigma-Aldrich) or Dulbecco's Modified Eagle Medium (DMEM; Lonza, Slough, UK) supplemented with 4 mm l-glutamine, 4.5 g/l glucose, 10% (w/v) heat-inactivated fetal bovine serum (Sigma-Aldrich), 100 U/ml penicillin and 100 µg/ml streptomycin (Sigma-Aldrich). Cells were grown at 5% CO_2_ and 37˚C.

### Immunoblotting

For whole cell extracts, cells were collected at 400 *g* for 3 min and washed with phosphate-buffered saline. Cells were re-suspended in lysis buffer (62.5 mm Tris-HCl, pH 6.8, 10% (v/v) glycerine, 2% (w/v) sodium dodecyl sulfate (SDS), 1 mm phenylmethylsulfonyl fluoride, 1 μg/ml pepstatin A, 1 μg/ml leupeptin, and 5 μg/ml aprotinin) and heated at 95 °C for 20 min. Protein content was determined with the Pierce Micro-BCA protein assay (Pierce, Northumberland, UK). An equal amount of protein (20 μg) was loaded onto SDS-polyacrylamide gels. Proteins were separated at 100 V for 2.5 h and then blotted to nitrocellulose membranes (Protean BA 83; 2 μm; Schleicher & Schuell) in transfer buffer (25 mm Tris, 192 mm glycine, 20% methanol (v/v), and 0.01% SDS) at 18 V for 60 min. The blots were blocked with 5% non-fat dry milk in Tris-buffered saline with Tween 20 (TBST) (15 mm Tris-HCl, pH 7.5, 200 mm NaCl, and 0.1% Tween 20) at room temperature for 1 h. Membranes were incubated with the primary antibodies at room temperature for 2 h or overnight at 4˚C. Membranes were washed with TBST three times for 5 min and incubated with peroxidase-conjugated secondary antibodies (Jackson Laboratories) for 1 h. Blots were washed and developed using the enhanced chemiluminescence detection reagent (Millipore, Ireland).

### Preparation of cell pellets for IHC

Cells were grown to a confluence of 50–75%. Cells were then detached and suspended in 10% phosphate-buffered formalin at room temperature and fixed for 4–6 h. Fixed cells were centrifuged at 500 × *g* for 3 min, washed once with 1 × PBS and pelleted again. A 1% agarose solution was prepared in 1 × PBS and cooled down to 40 °C in a water bath. The cell/agarose mixtures were transferred into plugs and let solidify. The agarose plugs were processed into paraffin blocks using standard tissue processing. Cell pellet samples (typically 0.6 mm in diameter) were then used for analysis.

### Tissue microarrays (TMAs)

TMAs of formalin-fixed paraffin-embedded (FFPE) tumour samples derived from 74 melanoma patients treated with Dacarbazine (alone or in combination with cisplatin or carboplatin), were generated. The TMA contained duplicate cores obtained from 14 primary melanomas, 62 metastatic melanomas and adjacent normal tissue. Demographics, clinical and follow-up information were available for the entire cohort. A total of *n* = 58 samples, representing untreated metastatic melanoma patients, were analysed for this study (Table 1).

**Table 1:** Summary of demographics and clinical information of the patients included in the study.

Characteristics		
Gender	*Value*	*%*
Male	30	51.7
Female	28	48.3
*Age at surgery (years)*	*Value*	*%*
< 65	44	75.9
≥ 65 and < 75	8	13.8
> 75	6	10.3
*Metastatic melanoma location*	*Value*	*%*
Distant skin site	10	17.2
Distant organ	17	29.3
Distant lymph node	28	48.3
Distant subcutaneous site	3	5.2
*Metastasis stage*	*Value*	*%*
M1a	8	13.8
M1b	8	13.8
M1c	42	72.4
*Primary melanoma type*	*Value*	*%*
Cutaneous	46	79.3
Mucosal	1	1.7
Ocular	2	3.4
Unknown	9	15.5
*Treatment*	*Value*	*%*
Dacarbazine	3	5.2
Dacarbazine, Cisplatin	54	93.1
Dacarbazine, Carboplatin	1	1.7
*Overall survival*	*t*_*0*_ *=* *sample collection*	*t*_*0*_ *=* *chemotherapy start*
Median (range) in months	19 (2–126)	11 (0–87)
*Progression-free survival*	*t*_*0*_ *=* *sample collection*	*t*_*0*_ *=* *chemotherapy start*
Median (range) in months	10 (1–100)	4 (0–83)

### Immunohistochemistry

IHC staining on FFPE cell pellets and tissue microarrays (TMA) was performed using an automated IHC platform (Link-48, Dako, Glostrup, Denmark) according to the manufacturer’s instructions. Sections (4 µm in thickness) were deparaffinised and antigen retrieval was performed at 95 °C for 15 min in appropriate buffer (high pH buffer, pH 9.0; low pH buffer, pH 6.0) using the PT-Link module (Dako, Glostrup, Denmark). A polymer-based detection system (EnVision Flex, Dako) was used with Permanent Red as the chromogen, resulting in a red colour endpoint that contrasted well with brown melanin. Sections were counterstained with haematoxylin. Positive and negative controls (omission of the primary antibody and replacement with the IgG-2a isotype control, mouse-ab18443; IgG isotype control, rabbit-ab208334, Abcam, Cambridge, UK) were included in each run. In addition, a Haematoxylin and Eosin (H&E) staining was performed for all slides of the TMAs, enabling pathologists to check for TMA core integrity, quality and tumour content.

### Core quality assessment

A pathological review of the H&E-stained sections and TMA blocks was conducted to define the quality of individual tissue cores and to assess the percentage of tumour tissue in each core. Each core was individually observed to determine whether there were any tissue artifacts (poorly fixed tissue, folded tissue, no tumour present, no tissue present, foreign material introduced at embedding, poor tissue microscopic details) or staining artifacts (knife marks across section, holes, clumps of stain precipitate, air bubbles), which would have compromised either the manual or automated image analysis. All quality assessments were independently validated by a second pathologist. Cores with compromising artifacts or with insufficient percentage of tumour cells were excluded from further analyses.

### Manual and automated scoring

IHC materials were first viewed at low power to judge overall quality and distribution of staining. Subsequently, staining frequency (total % stained cells) and staining intensity (intensity of stained cells; 0 = no staining, 1+ = weak staining, 2+ = moderate staining, 3+ = strong staining) were determined. Histoscores (*H* scores) were then calculated as follows:

$$H {\mathrm{score}} = 1 \times \% _{cells\;1 + } + 2 \times \% _{cells\;2 + } + 3 \times \% _{cells\;3 + }$$

The manual scoring was performed on images acquired with the Aperio ScanScope XT slide scanner (Aperio Technologies, Vista, CA) used at × 20 magnification with a maximum pixel resolution of 0.5 µm. ImageScope analysis software (Aperio Technologies, Vista, CA) was used for viewing and analysing digital images. Aperio Spectrum software was used to generate individual tissue spot images for automated analysis. The Colour Deconvolution algorithm (Aperio Technologies) was used to obtain quantitative values for average positive intensity (average intensity of pixels positively stained, graded from 0, 1, 2, 3) and total percent positive (percentage of positive stained area in relation to total area of the core). Histoscores were calculated as described above.

### Survival analysis

PFS was calculated as the time between the surgery that procured the sample and the date of disease progression or of a new metastatic event in a different location. Pathologist’s and automated *H* score were used to separate patients with high (above median) and low (below median) expression of each marker protein included in this study. In case more than one tissue core with satisfactory quality was available for a single patient, the average *H* score was considered. Log-rank testing was used to compare the two groups over a follow-up time of 36 months. Log-rank testing for trends was used when comparing three groups. Kaplan–Meier survival curves were generated and compared using GraphPad Prism (version 4.03). For analysis of data stored in the cancer genome atlas (TCGA), normalised mRNA expression data (upper quartile normalised Fragments per Kilobase of transcript per million mapped reads, log_2_(FPKM-UQ + 1)) generated by the Genomic Data Commons (GDC-NIH) were downloaded from the UCSC-XENA browser^25,26^. The SKCM cohort, unlike other TCGA data sets, contains mainly metastatic samples^27^ (370 out of 477), some of which were collected a long time after initial diagnosis of the primary melanoma^28^. In order to correlate mRNA expression to progression of metastatic disease, the ‘*new tumour event free survival*' was calculated as the time between sample collection and the first new tumour event (in case of multiple new tumour events during the follow-up time) or, in case of no new tumour events, death. If a new tumour event was reported before the date of sample collection, the patient was excluded from the sub-cohort. Follow-up data and associated clinical records were downloaded from Broad GDAC Firehose^29^ (new tumour event time from initial diagnosis) and UCSC-XENA browser^25,26^ (overall survival from initial diagnosis), respectively. Sample collection information are available through the GDC data portal^30,31^ (time from initial diagnosis to sample collection). As treatment information are not routinely available for all deposited metastatic melanoma cases, we downselected the cohort to stage III/IV melanoma patients diagnosed with metastatic melanoma before 2010, to ensure that chemotherapy-based treatment options would have been the standard first line of treatment (*n* = 79 patients). An optimised chi-square-based cutoff was determined to divide patients with high and low *BAX*, *BAK1* and *DIABLO* (Smac) mRNA amounts, and the two groups were compared by log-rank test. The cutoff for each marker was obtained by selecting the cohort separation that resulted in the highest chi-squared value with the function *survdiff* of the library *survival* in R (version 3.4.0). Median cutoff-based results are reported in Supplementary Fig. 4. Log-rank test for trend was used when comparing three groups. Kaplan–Meier survival curves were generated and compared using GraphPad Prism (version 4.03).

### Data-driven modelling and pattern recognition

A data-driven modelling approach based on a previously published method^32^ was developed to predict patients’ PFS using *H* scores generated by automated image analysis as input. The pipeline was developed for MATLAB (version 2016a, The Mathworks, UK), equipped with the statistical toolbox. Prior to the analysis, patients with a complete protein panel (*n* = 50) were divided into two PFS categories: PFS > 12 months (*n* = 17) and PFS < 12 months (*n* = 33). After standardisation of the initial data set, a principal component analysis (PCA) was performed and the principal components (PC) with an eigenvalue > 1 were considered for subsequent analyses. The patients were positioned in the 3D space defined by the first three PCs according to the scores computed by PCA, and linear discriminant analysis (LDA) was used to test the class segmentation accuracy. To evaluate the predictive potential of the framework, leave one out cross validation (LOOCV) followed by LDA was applied iteratively to the data set, using 49 patients as training set and one patient as test at each iteration. LDA was also applied to a data set reduced to three proteins (Bax, Bak and Smac), skipping the initial dimensionality reduction step. PCA and LDA were performed using the functions *pca* and *classify*, respectively. The predictive performance of the two classification models was compared by computing the area under the curve (AUC) with the function *perfcurve*.

## Results

### Low expression of pro-apoptotic proteins Bax, Bak and Smac correlates with increased PFS in chemotherapy-treated metastatic melanoma

**Fig. 1:**
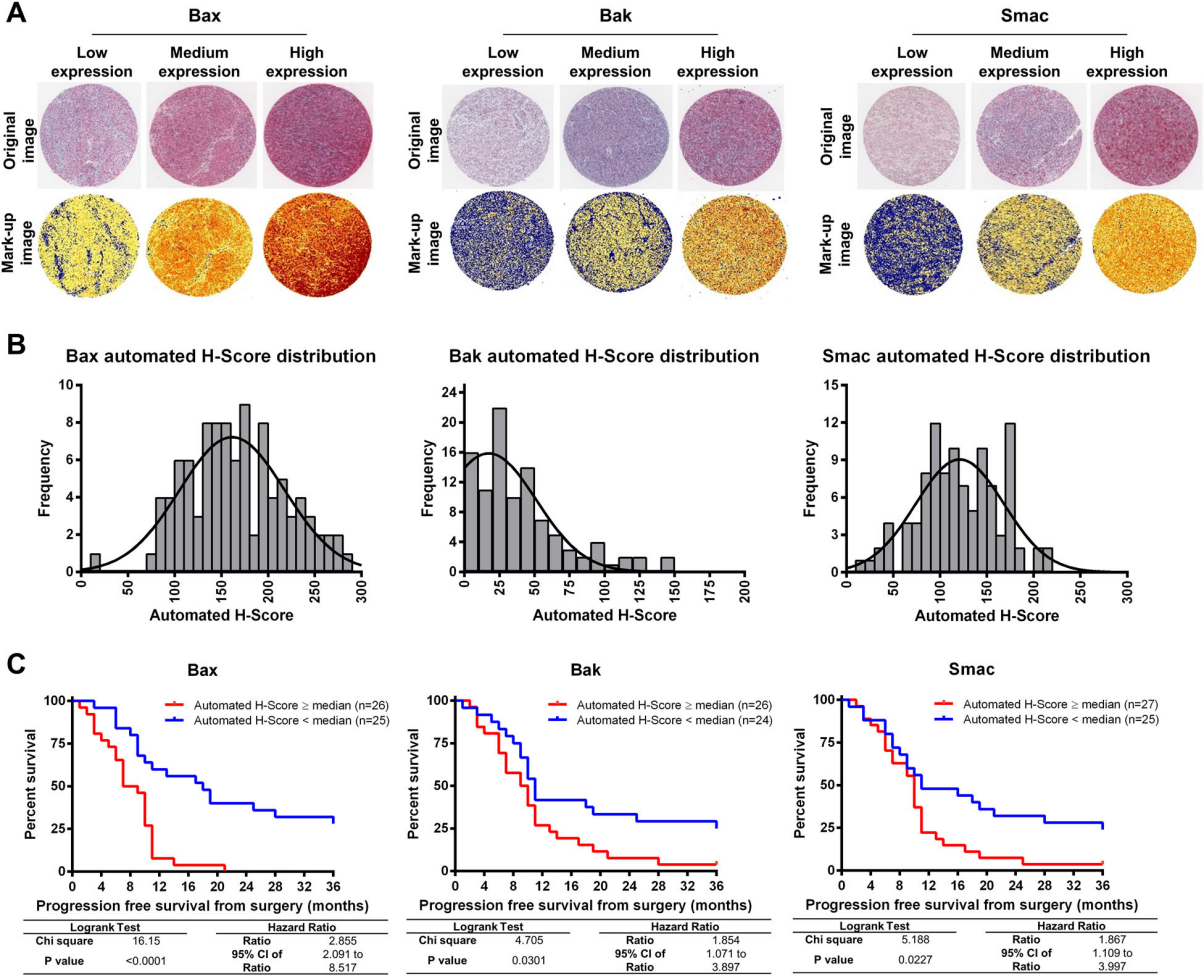
Low expression of pro-apoptotic proteins Bax, Bak and Smac correlates with increased progression-free survival (PFS) in chemotherapy-treated metastatic melanoma. **a** Tissue cores stained by IHC for Bax, Bak and Smac. Representative original (left panels) and mark-up images (right panels) of cores with low, medium and high expression of the three proteins are shown. The mark-up images were quantified to compute automated *H* scores. Table insert shows cohort information. **b** Distribution of *H* scores across the analysed melanoma tissue cores. Only stained cores that passed the quality control were retained for subsequent analyses (Bax *n* = 100 cores from 52 patients, Bak *n* = 100 cores from 51 patients, Smac *n* = 104 cores from 53 patients). **c** Survival analysis based on *H* scores for Bax, Bak and Smac. Median *H* scores were used as cutoff to separate the patients with high (red line) and low (blue line) expression of each protein. Log-rank test was used to compare the Kaplan–Meier curves for progression-free survival from the date of sample procurement.

**Fig. 2:**
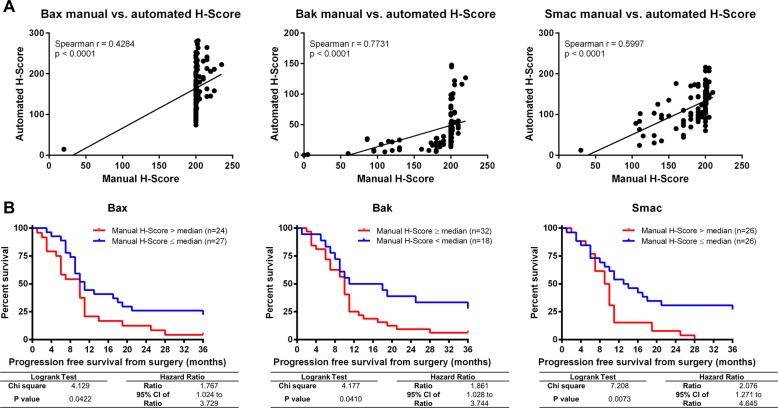
Manual scoring confirms association of low Bak, Bax and Smac protein expression with improved PFS. **a** Correlation between automated and manual *H* scores. Manual scores were obtained from two independent pathologists blinded to patient follow-up. Correlation was analysed using Spearman’s rank correlation coefficient. **b** Median *H* scores were used as cutoff to separate the patients with high (red line) and low (blue line) expression of each protein. Log-rank test was used to compare the Kaplan–Meier curves for progression-free survival from the date of sample procurement.

Genotoxic chemotherapy based on DNA-alkylating agents such as dacarbazine induces intrinsic apoptosis, preferentially in proliferating cells such as cancer cells. Intrinsic apoptosis is governed by the family of Bcl-2 proteins and the subsequent signalling network of the apoptosis execution phase. We therefore tested key players of this apoptosis signalling modules as potential prognostic markers in metastatic melanoma. In total, we analysed the expression of six pro-apoptotic (Bax, Bak, Smac, Procaspase-9, Apaf-1, Procaspase-3) and three antiapoptotic proteins (Bcl-2, Bcl-xL, XIAP) in metastatic melanoma samples spotted on TMAs. Only treatment-naive samples from metastases were used for subsequent analyses. Information such as patient demographics, histopathology and staging, treatment and follow-up are provided as Supplementary Table 1 and are summarised in Table 1. Following comprehensive antibody validation (Supplementary Fig. 1A–D), IHC stains for *n* = 58 tumour metastases matching the inclusion criteria were analysed from the TMAs. Only tissue samples passing independent pathologist quality control for tissue integrity and staining artifacts were considered for subsequent analyses (Supplementary Table 2). TMAs scans were then used to generate mark-up images of the tissue cores, followed by automated quantification of staining intensities (see methods). The dynamic range of the staining intensities allowed to confidently define quartiles of negative, low, medium and high staining for protein expression (see Fig. 1a for examples for Bax, Bak and Smac). From these, *H* scores were calculated for each tumour sample (Fig. 1b), thereby allowing comparison with best practice manual scoring (see Fig. 2). To test if protein expression amounts and patient prognosis correlate, we performed survival analyses for all nine apoptosis regulatory proteins. Kaplan–Meier curves representing PFS from the date of sample procurement showed that low amounts of pro-apoptotic proteins Bax, Bak and Smac significantly correlated with better prognosis (Fig. 1c). With the exception of Procaspase-9, which associated with better prognosis in this analysis, none of the other proteins (Bcl-2, Bcl-xL, Apaf-1, XIAP and Procaspase-3) individually correlated with better or worse prognosis (Supplementary Fig. 2). Overall, these results surprisingly indicate that low amounts of apoptosis-inducing proteins Bax, Bak and Smac are linked to a better prognosis in chemotherapy-treated metastatic melanoma.

### Manual scoring confirms association of low Bak, Bax and Smac protein expression with improved PFS

To further validate our findings, we next conducted best practice manual scoring of the stained TMAs. *H* scores for all marker candidates were obtained from two independent pathologists, both blinded to patient PFS. Plotting *H* scores obtained by automated analysis against manual *H* scores, we noted that manual scores strongly clustered at values of ~ 200, whereas automated scoring provided higher granularity across the entire dynamic range (Fig. 2a, Fig. 1b, Supplementary Table 2). This highlights that manual scoring appears limited in differentiating within the range of medium staining intensities and frequencies. Nevertheless, median separation of patient samples based on manual *H* scores provided survival curves for Bax, Bak and Smac staining that were very similar to those obtained by automated scoring (Fig. 2b). In contrast, the manual scores for all other proteins failed to separate patients with high and low PFS (Supplementary Fig. 3). These results therefore demonstrate that the Bax, Bak and Smac signatures are robust enough to also be captured in routine manual IHC-based biomarker discovery workflows.

### Combined low expression of Bax, Bak and Smac is a combinatorial marker candidate for improved PFS

**Fig. 3:**
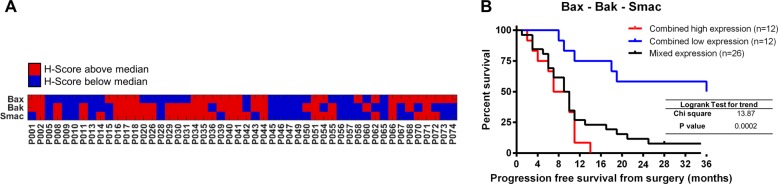
Combined low expression of Bax, Bak and Smac is a combinatorial marker candidate for improved progression-free survival. **a** Expression profiles based on the *H* scores of Bax, Bak and Smac (blue: automated *H* score below median, red: above median) in *n* = 50 patients. **b** Survival analysis for the cohort based on the expression profiles shown in a. Log-rank test for trend was used to compare three Kaplan–Meier curves representing patients with low Bax, Bak and Smac *H* scores (blue) vs. mixed and high expression (black and red, respectively).

During apoptosis, Bax and Bak form pores in the outer mitochondrial membrane, leading to Smac release into the cytosol. Owing to the significant correlation of the single proteins with PFS and their direct relationship within the apoptosis signal transduction cascade, we checked if combinations of the three markers could improve prognostication of PFS. For the *n* = 50 patients for which *H* scores for Bax, Bak and Smac were available, we noted that combined low or high staining for all three markers was restricted to subsets of the tumour samples (Fig. 3a). We therefore divided the cohort into three groups of combined high expression, heterogeneous expression and combined low expression. PFS-based survival analysis of the three groups demonstrated that patients harbouring tumours with combined low expression of Bax, Bak and Smac showed significantly improved PFS, extending beyond 36 months for 50% of this subgroup (Fig. 3b). In contrast, when only one or two markers where expressed in low amounts, PFS improved only slightly (median PFS = 10 months vs. 8.5 months when all three markers were highly expressed) (Fig. 3b). Overall, this shows that Bax, Bak and Smac could jointly define a signature that strongly associates with PFS, with combined low expression indicating improved PFS.

### TCGA-SKCM-based analysis validates the prognostic Bax, Bak, Smac signature

**Fig. 4:**
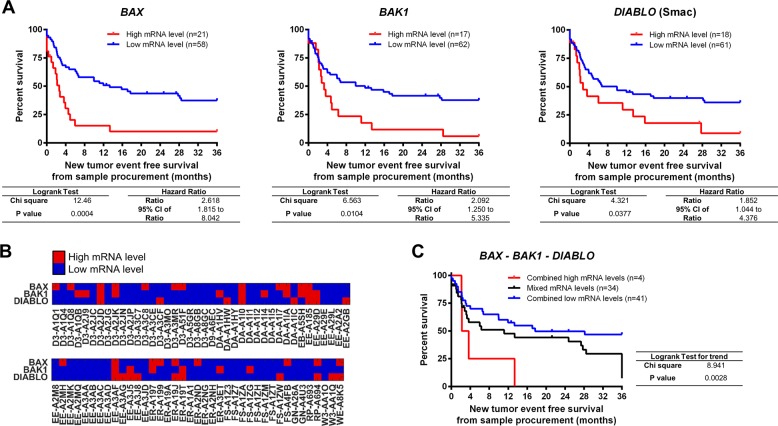
TCGA-SKCM-based analysis validates the prognostic Bax, Bak, Smac signature. Independent validation of the prognostic signature at transcriptome level in the SKCM-TCGA metastatic sub-cohort. **a** Survival analysis in the SKCM-TCGA sub-cohort (*n* = 79 patients diagnosed with stage III or IV metastatic melanoma before 2010). An optimised chi-square-based cutoff was determined to divide patients with high (red) and low (blue) normalised *BAX*, *BAK1* and *DIABLO* (Smac) expression (log_2_(FPKM-UQ + 1)). Kaplan–Meier curves (follow-up from sample procurement) were compared by log-rank test. **b** mRNA amounts for *BAX*, *BAK1* and *DIABLO* (Smac) (blue: mRNA level below cutoff, red: mRNA level above cutoff). **c** Survival analysis in the metastatic TCGA-SKCM sub-cohort based on the expression profiles in Fig. 4b. Log-rank test for trend was used to compare three Kaplan–Meier curves representing patients with combined low *BAX*, *BAK1* and *DIABLO* (Smac) expression (blue), combined high expression (red) or with mixed expression (black).

To independently validate the prognostic potential of Bax, Bak and Smac expression, we analysed transcriptome data of *n* = 79 metastatic melanoma patients from the TCGA-SKCM cohort (Table 2). The survival analysis revealed that low *BAX*, *BAK1* and *DIABLO* (*SMAC*) mRNA amounts significantly correlate with better prognosis (Fig. 4a). As previously observed at protein level, the expression pattern between *BAX*, *BAK1* and *DIABLO* was heterogeneous across the cohort (Fig. 4b). Patients with low tumour mRNA amounts across all three markers had a significantly better prognosis than patients in which at least one marker was highly expressed (Fig. 4c). Taken together, these results recapitulate in an independent cohort the trends observed at protein level, confirming the prognostic potential of Bax, Bak and Smac as a combinatorial marker in chemotherapy-treated metastatic melanoma.

**Table 2:** Patient demographics and clinical information of the metastatic SKCM-TCGA sub-cohort.

Characteristics		
*Gender*	*Value*	*%*
Female	32	40.51
Male	47	59.49
*Ethnicity*	*Value*	*%*
White (non-Hispanic or Latino)	79	100
*Disease stage at initial diagnosis*	*Value*	*%*
Stage III	68	86.08
Stage IV	11	13.92
*Age at diagnosis (years)*		
Mean	55.5	
Median	55	
Range	18–87	
*Overall survival from initial diagnosis (months)*		
Mean	35.1	
Range	2.6–175.2	
*Overall survival from sample procurement (months)*		
Mean	15.9	
Range	1.1–64.9

### Pattern recognition allows predicting patient prognosis

**Fig. 5:**
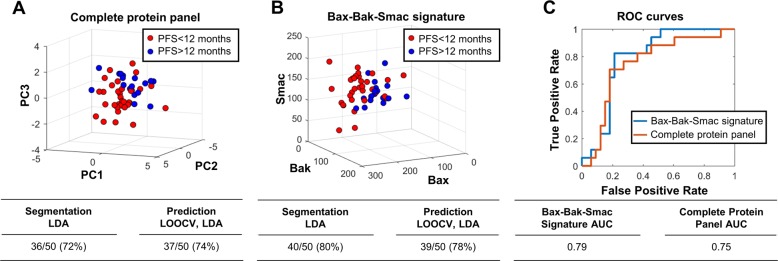
Pattern recognition allows predicting patient prognosis. **a** A principal component analysis was performed on the *H* scores of nine apoptotic proteins. To provide a visualisation of the spatial clustering, patients samples were positioned in a 3D scatter plot defined by the first three principal components and colour-coded according to their PFS time (red < 12 months, *n* = 33; blue > 12 months, *n* = 17). Linear discriminant analysis (LDA) correctly segmented 72% of the patients. Leave One Out Cross Validation (LOOCV) combined with LDA predicted the correct class for 74% of the patients. **b** 3D scatter plot showing the spatial clustering of patients with short and long PFS based on the *H* scores for Bax, Bak and Smac. LDA correctly segmented 80% of the patients and LOOCV-LDA achieved 78% prediction accuracy. **c** Comparison of the performance of the two classifiers shown in **a** and **b**. The receiver operating characteristic curves (ROCs) and respective areas under the curve (AUC) are shown.

We next applied a data-driven pattern recognition approach to study if the Bax, Bak, Smac signature would be sufficiently strong to predict patient PFS from protein expression profiles^32^. First, *H* scores from automated TMA analysis for all marker candidates were subjected to a PCA (applied to the 50 patient samples for which the complete nine protein panel was available). Patient tumours were positioned into a 3D space defined by the first three PCs and colour-coded to represent high or low PFS (PFS > 12 months and PFS < 12 months). Visually inspecting the scatter plot, we noticed a tendency of patients with high or low PFS to occupy distinct sub-regions of the PC space (Fig. 5a). To objectively assess the quality of this segregation, we applied LDA. LDA segmentation encouragingly separated 72% of the patients into their correct prognosis sub-space. Next, we tested if these patterns were sufficiently strong to also predict the PFS category on a case-by-case basis. To do so, we performed leave one out cross validation (LOOCV). At each iteration, the PFS category of one patient was predicted after using the remaining 49 patients as a training set that defined the PCA subspaces for high and low PFS. The panel of nine apoptosis regulatory proteins allowed to correctly predict high or low PFS in 74% of patients. Since our previous survival analyses (Fig. 1) showed that only Bax, Bak and Smac consistently correlated with PFS, we likewise tested if a similarly good or even better performing classifier can be derived from those three markers alone. Indeed, cluster segmentation and prediction accuracy tended to improve to 80% and 78% accuracy, respectively (Fig. 5b). In conclusion, as highlighted by the comparison of receiver operating characteristic (ROC) curves (Fig. 5c), classification based on the Bax-Bak-Smac signature alone is sufficient to obtain high prediction accuracies for patient PFS, whereas the remaining protein markers do not carry meaningful information to improve these predictions. Overall, this strengthens the evidence for low Bax, Bak and Smac expression being associated with better prognosis in metastatic melanoma and points out a route by which pattern recognition allows generating predictions for patient prognosis.

## Discussion

Apoptosis is the major cell death modality by which anticancer therapies eliminate malignant neoplastic cells. In this study, we assessed if proteins that regulate the two major apoptosis decision hubs, namely the apoptotic engagement of mitochondria and the terminal execution phase of apoptosis^33^, alone or in combination can serve to prognosticate PFS in metastatic melanoma patients undergoing dacarbazine-based chemotherapy. We found that low rather than high expression of the pro-apoptotic proteins Bax, Bak and Smac correlates with higher PFS, and that these three proteins in combination can serve as a combinatorial prognostic marker with a promising AUC of 0.79.

Owing to the central role of apoptosis in tumour cell elimination, the finding that low expression of pro-apoptotic proteins correlated with better prognosis in metastatic melanoma contradicted our expectations. However, counter-intuitive relationships between the expression patterns of apoptosis inducers or antiapoptotic genes or proteins were reported previously. For example, high expression of Bax was found to correlate with an increased risk for relapse in childhood acute lymphoblastic leukaemia^34^. High expression of Bax, measured as transcript and protein amounts, respectively, has also been associated with poor prognosis in acute myeloid leukaemia and non-Hodgkin lymphoma^35,36^. Similarly, studies in which high expression of the Bax antagonist Bcl-2 has been reported to correlate with better prognosis can be found for colorectal, breast, glioma, gastric and non small cell lung cancer^37–43^. Bax and Bcl-2 are the best-characterised members of the Bcl-2 protein family, which controls mitochondrial engagement in apoptosis signal transduction^44,45^, whereas Bak has been less thoroughly studied. Bak functions as a Bax-like protein and upon activation likewise is able to form pores in the outer mitochondrial membrane, thereby triggering apoptosis execution^44,45^. Links between low Bak expression and an improved outcome have not been reported in metastatic melanoma so far, but reduced *BAK* mRNA amounts were associated with better overall survival in hepatocellular carcinoma^46^. Similarly, a counter-intuitive prognostic value of Smac has not yet been reported in melanoma, but high expression was found to correlate with early local disease recurrence in cervical cancer^47^. It needs to, however, be stated that in reverse a large body of literature associates high expression of pro-apoptotic or a low expression of antiapoptotic genes or proteins with better outcome, as would intuitively be expected (see e.g., ref. ^48–59^). Overall, it therefore appears that signatures indicative of apoptosis competency or resistance need to be interpreted or studied within the specific disease setting and context. For example, it was suggested that expression patterns indicative of high apoptosis responsiveness may correlate with poor outcome if dormant, stem-like cancer cells that may reside within tumour tissues re-populate tumours and promote further spread and progression of the disease after the bulk population of cells has been eliminated by apoptosis-inducing therapy^42,43^. In line with this, apoptotic cell loss can drive the proliferation of surrounding cells, for example, through caspase-dependent prostaglandin signalling and secretion of other proliferation stimulating factors from dying cells^60,61^. These signalling processes indeed might be of relevance in melanoma treatment responsiveness and disease relapse^62^.

As apoptosis resistance is a hallmark of cancer^63^, it nevertheless appears puzzling that reduced expression of apoptosis drivers correlates with better prognosis in a treatment scenario that is clearly geared towards apoptosis induction. In addition to the above line of thoughts, the very high mutation burden of cutaneous melanoma^64^ might provide the basis for an additional explanation. Although unfavourable expression of key apoptosis regulators in many cases may cause apoptotic cell death to be suppressed during cell transformation, tumour development and disease progression, and as such could be considered causative for the disease, such low basal apoptosis susceptibilities might nevertheless be overcome by elevated apoptosis-inducing stress in chemotherapy settings. In contrast, where low apoptosis susceptibility is not causative for the disease (and hence protein expression profiles would indicate “normal” susceptibility), other alterations and mutations might drive the development and progression of the disease. Many of these could prevent therapy-induced stress signals to be channelled towards apoptosis induction. Indeed, low expression of Bax and Bak might be linked to disease progression in earlier stages of melanoma. Although Bax protein expression tends to be higher in melanoma tissues than in benign nevi^65^, low expression of Bax within primary superficial-spreading melanoma was associated with poor prognosis and therefore could indicate a role in disease development and progression^66^. Similar findings were reported for Bak expression in the same study. Also in stage IIa melamoma, low Bax and Bak protein expression was associated with poor prognosis, with the majority of such patients developing metastatic disease^67^. Taken together, these prior reports combined with our findings therefore suggest that low expression of pro-apoptotic players could be causative for early stage tumour formation and melanoma disease progression by lowering basal apoptosis susceptibility, and that this reduced susceptibility can be overcome once pro-apoptotic stress is elevated externally, for example by dacarbazine-based chemotherapy. It will be interesting to see if similar relationships can also be found in other cancer (sub)types in the future.

## Supplementary information

Supplemental figures and tables legends

Supplemental figure 1

Supplemental figure 2

Supplemental figure 3

Supplemental figure 4

Supplemental table 1

Supplemental table 2
