# Supplementary material for: Low expression of pro-apoptotic proteins Bax, Bak and Smac indicates prolonged progression-free survival in chemotherapy-treated metastatic melanoma
Source: Cell Death Dis. 2020 Feb 13;11(2):124. doi: 10.1038/s41419-020-2309-3 (PMC7018795; doi:10.1038/s41419-020-2309-3)
Supplement: Supplementary file 1 — Supplemental figures and tables legends [file 41419_2020_2309_MOESM1_ESM.docx]

**Supp. Fig.1 Antibody validation**

Commercially available antibodies for all markers to be studied were tested for specificity by Western blotting and by IHC on cell line pellets. Only results for antibodies passing quality assessment are shown. In Western blotting experiments, cell lines with known high or low/absent expression of the marker of interest were compared. Antibodies were required to yield single or clearly dominant specific bands at reported apparent molecular weights of marker proteins and/or their splice variants. IHC-based validation were required to clearly differentiate between cell line pellets with high or low/absent expression of the markers and to yield homogeneous staining across the isogenic populations. Subsequently, stained full face tissue sections were assessed by pathologists before staining protocols were approved for TMA stainings. **a** Validation results for anti-apoptotic Bcl-2 family members Bcl-2 and Bcl-xL; **b** Validation results for pro-apoptotic Bcl-2 family members Bax and Bak; **c** Validation results for Smac and its antagonist XIAP; **d** Validation results for Apaf-1 and the zymogens of caspases-9 and -3.

**Supp. Fig.2 Survival analysis for the cohort based on automated H-Score of Bcl-2, Bcl-xL, XIAP, Apaf-1, Procaspase-9 and Procaspase-3**

Survival analysis based on automated H-Scores for Bcl-2, Bcl-xL, XIAP, Apaf-1, Procaspase-9 and Procaspase-3. Median H-Scores were used as cut-off to separate the patients with high (red line) and low (blue line) expression of each protein. Log-rank test was used to compare the Kaplan-Meier curves for progression free survival from the date of sample procurement.

**Supp. Fig.3 Survival analysis for the cohort based on manual H-Score of Bcl-2, Bcl-xL, XIAP, Apaf-1, Procaspase-9 and Procaspase-3**

Survival analysis based on manual H-Scores for Bcl-2, Bcl-xL, XIAP, Apaf-1, Procaspase-9 and Procaspase-3. Median H-Scores were used as cut-off to separate the patients with high (red line) and low (blue line) expression of each protein. Log-rank test was used to compare the Kaplan-Meier curves for progression free survival from the date of sample procurement.

**Supp. Fig.4 Survival analysis in the metastatic TCGA-SKCM sub-cohort** Survival analysis based on *BAX*, *BAK1* and *DIABLO* (Smac) mRNA expression. Median normalized mRNA amount (log_2_(FPKM-UQ+1)) was used as cut-off to separate the patients with high (red line) and low (blue line) expression of each transcript. Log-rank test was used to compare the Kaplan-Meier curves for progression free survival from the date of sample procurement.

**Supp. Table 1** Patient demographics, histopathology and staging, treatment and follow-up information of the cohort.

**Supp. Table 2** IHC digital and manual image analysis results and cores quality control.
