# Supplementary figures and images for: Low expression of pro-apoptotic proteins Bax, Bak and Smac indicates prolonged progression-free survival in chemotherapy-treated metastatic melanoma
Source: Cell Death Dis. 2020 Feb 13;11(2):124. doi: 10.1038/s41419-020-2309-3 (PMC7018795; doi:10.1038/s41419-020-2309-3)

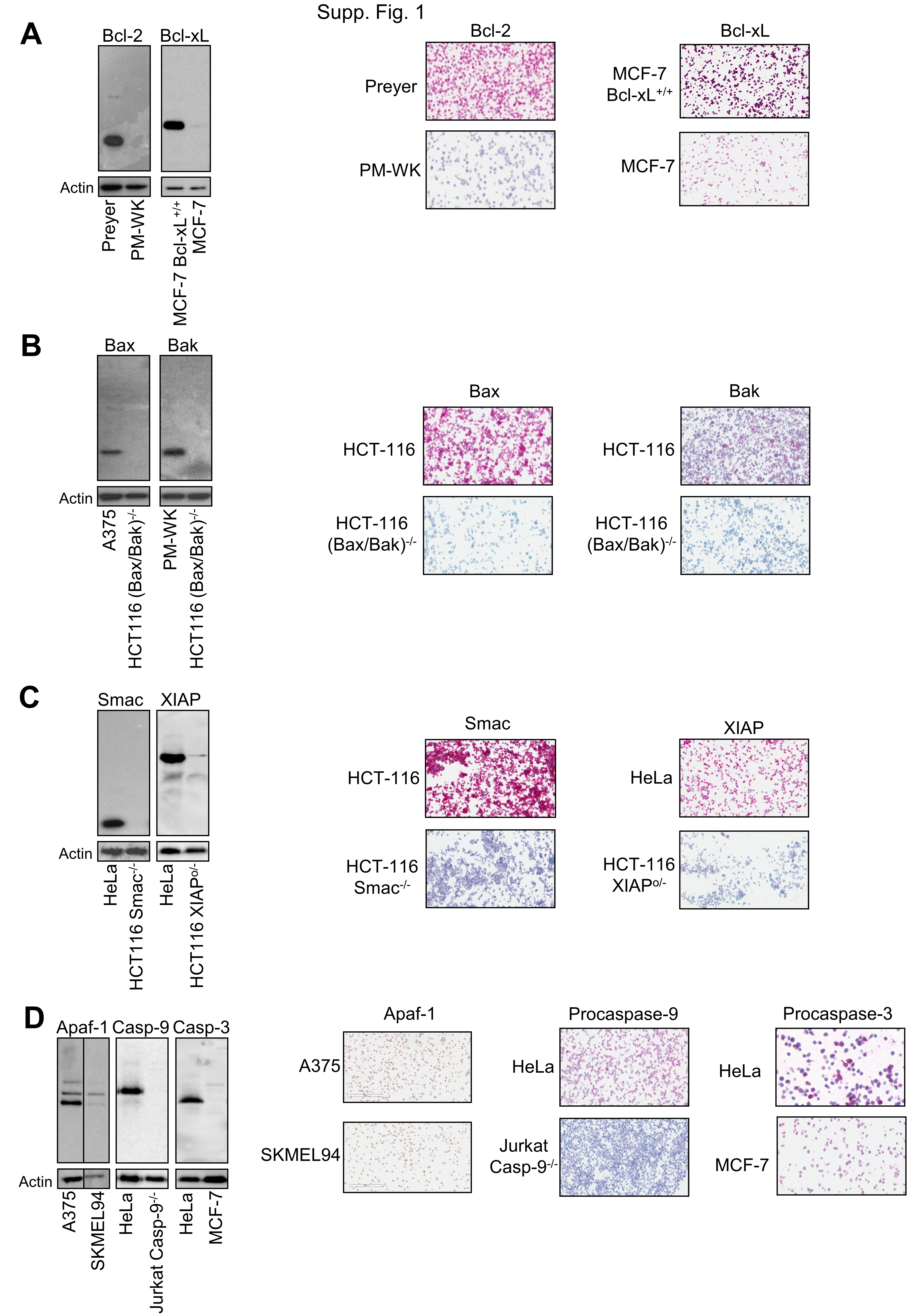

Supplement: Supplementary file 2 — Supplemental figure 1 [file 41419_2020_2309_MOESM2_ESM.tif]

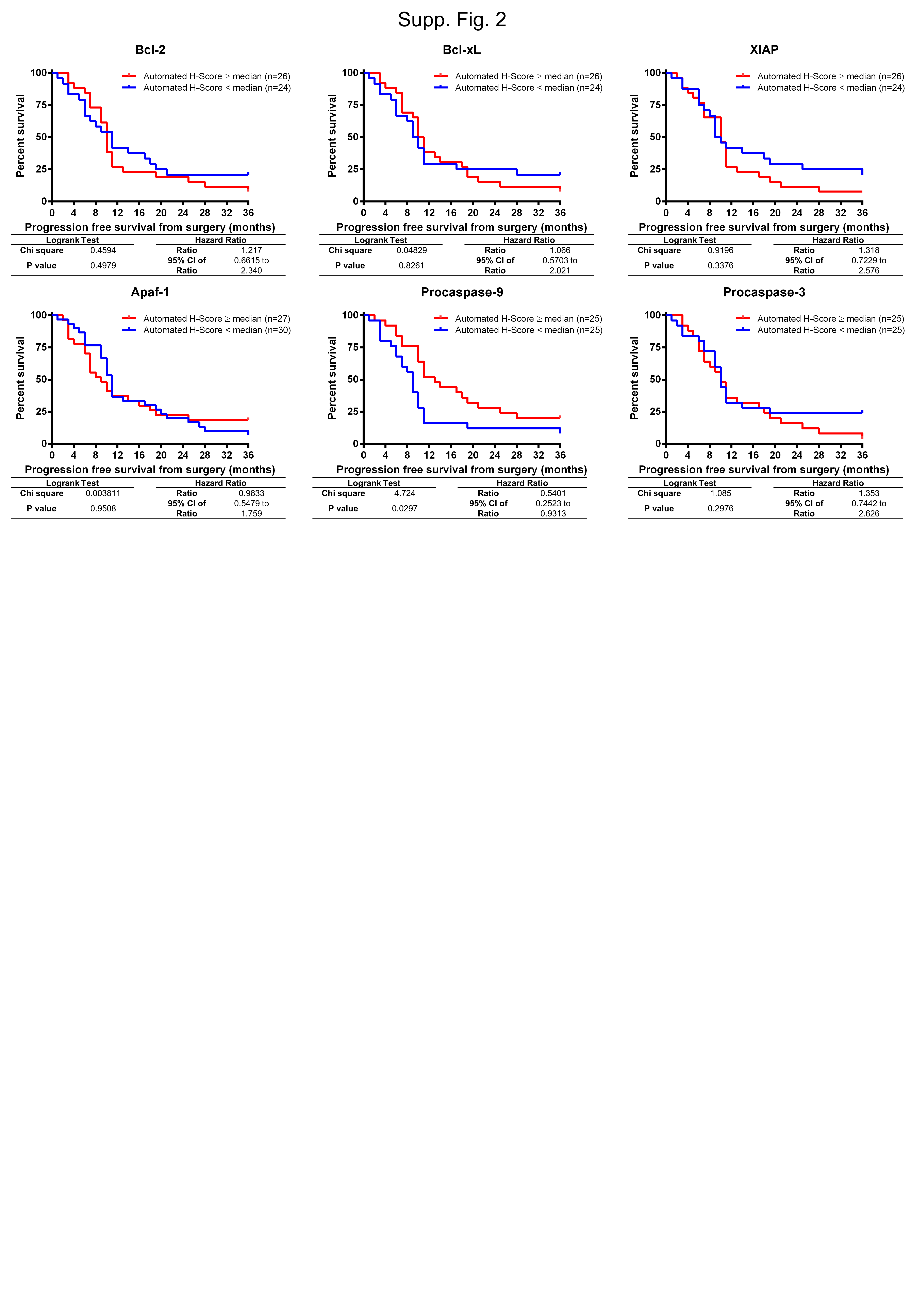

Supplement: Supplementary file 3 — Supplemental figure 2 [file 41419_2020_2309_MOESM3_ESM.tif]

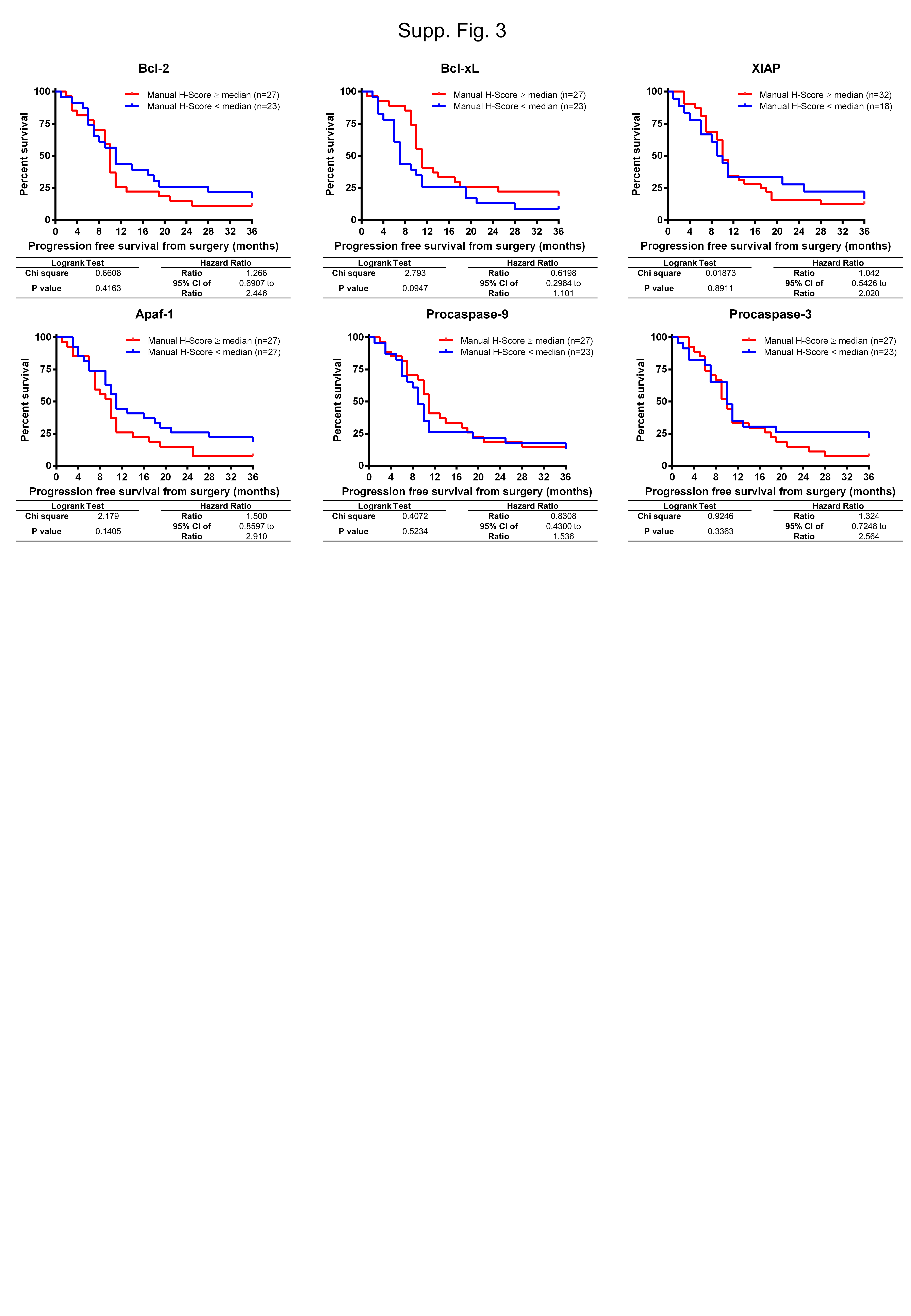

Supplement: Supplementary file 4 — Supplemental figure 3 [file 41419_2020_2309_MOESM4_ESM.tif]

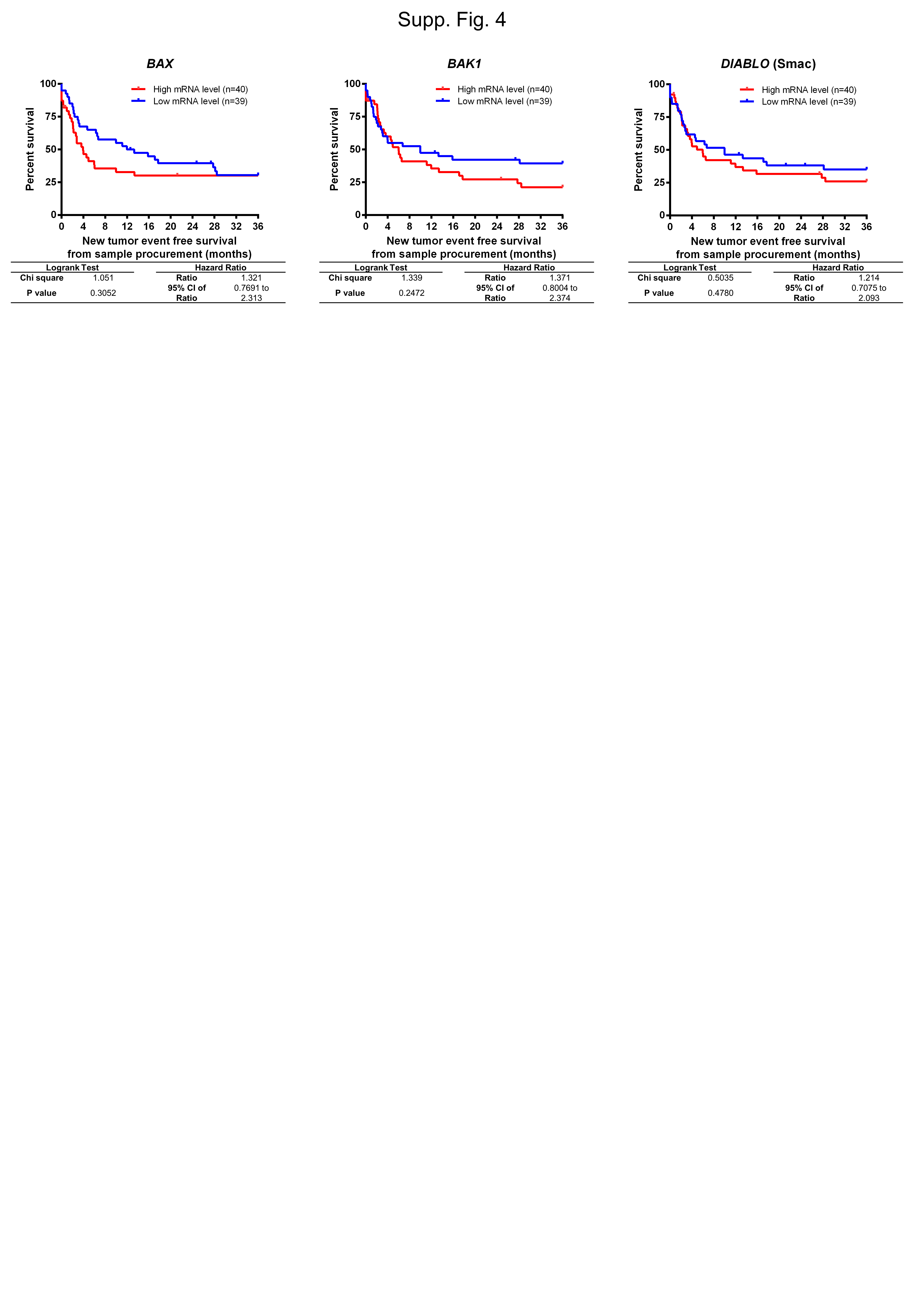

Supplement: Supplementary file 5 — Supplemental figure 4 [file 41419_2020_2309_MOESM5_ESM.tif]
